# Learning Effectiveness Assessment between Primary School Students and Adults in Basic Life Support Education

**DOI:** 10.1155/2021/5579402

**Published:** 2021-02-24

**Authors:** Ming-Fang Wang, Yi-Kan Wu, Cheng-Yu Chien, Li-Heng Tsai, Chen-Bin Chen, Chen-June Seak, Chi-Chun Lin, Chien-Hsiung Huang, Chung-Hsien Chaou, Hsiao-Jung Tseng, Chip-Jin Ng

**Affiliations:** ^1^Department of Emergency Medicine, Chang Gung Memorial Hospital, Linkou and College of Medicine, Chang Gung University, Tao-Yuan, Taiwan; ^2^Department of Emergency Medicine, Ton-Yen General Hospital, Zhubei, Taiwan; ^3^Graduate Institute of Business and Management, Chang Gung University, Taoyuan, Taiwan; ^4^Department of Emergency Medicine, New Taipei City Hospital, New Taipei City, Taiwan; ^5^Biostatistical Unit, Clinical Trial Center, Chang Gung Memorial Hospital, Linkou, Taiwan

## Abstract

**Background:**

Out-of-hospital cardiac arrest (OHCA) remains a big issue of critical care. It is well known that bystander cardiopulmonary resuscitation (CPR) with an automated external defibrillator (AED) used did improve the survival rate. Therefore, CPR education including basic life support (BLS) and AED has been advocated for years. It showed significant improvement of knowledge and willingness to perform CPR through adolescents after the course. However, little is known regarding the ability and learning effectiveness of school students who attend such courses. Therefore, this study aimed to evaluate the CPR effectiveness of both adolescents (12 years old) and adults who undergo the same course of BLS and AED.

**Methods:**

This is a retrospective study. Sixth-grade elementary school students in Northern Taiwan were selected to compare with the adult group. Both took 90 minutes of the BLS and AED course by the doctor with BLS instructor qualification. The primary outcomes were CPR quality and passing or failing the skill examination parameters. The secondary outcome was the posttraining written test and questionnaire of CPR willingness.

**Results:**

In the written test, there was a statistical difference in the pretest score except AED knowledge, but no difference was revealed in the posttest score. No statistical difference in CPR quality was noted. In the skill examination, only checking breathing status had statistical difference (elementary group (71%) vs. adult group (86%) (*p*=0.003)).

**Conclusion:**

We revealed that sixth-grade elementary students' performance in CPR and AED was similar to that of adults after completing the current 90-minute course. Therefore, we strongly advocate offering CPR and AED courses to 12-year-old children, and these courses should emphasize checking the victim's breathing status.

## 1. Introduction

Out-of-hospital cardiac arrest (OHCA) is a critical public health concern. OHCA has a lower incidence rate compared with other diseases; however, it has a high mortality rate. In the United States, the OHCA rate per 100,000 people is 3.5 [1]. Between 2000 and 2012 in Taiwan, approximately 51.1 people per 100,000 experienced OHCA [2]. According to a study conducted in Paris, up to 70% of OHCAs occur in residential areas, and 30% occur in public areas [3]. Moreover, a recent retrospective study in Taiwan with data from 2012 to 2016 revealed that 80% of OHCAs occur in private places [4]. A systematic review also reported that approximately 53% of events are witnessed by a bystander, and bystander cardiopulmonary resuscitation (CPR) remains low at 32% [5]. However, bystander CPR rates vary considerably worldwide, ranging from 10% to 65% in the United States [6]. Multiple factors are responsible for these large differences in the bystander CPR rate, including social economic status, racial and educational characteristics, and location of the collapse [7–9].

Bystander CPR with an automated external defibrillator (AED) is widely known to improve the survival rate. Moreover, bystander CPR significantly increases (by up to two- to four-fold) 30-day and 1-year survival regardless of witnessed status [10]. Nevertheless, according to related studies in Taiwan, bystander CPR rates before the arrival of an emergency medical technician (EMT) ranged from 17% in 2008 to approximately 30% between 2012 and 2016 [4, 11]. According to a US study, bystander CPR rates have increased slightly over time: from 28.2% in 2005-2006 to 36.3% in 2012 [12]. CPR education has been expanding for years, with considerable public health benefits; however, obstacles remain regarding the execution of CPR by the public. According to a study conducted in Taiwan, the main reasons people hesitate to perform CPR are fear of legal consequences (44%) and harming patients (36.5%) [13]. Concerning the legal aspect, the Good Samaritan Law was passed in 2013 in Taiwan to protect people against legal consequences if they perform CPR incorrectly on a stranger in a critical situation. In addition to legal protection, CPR educational training should also be promoted to increase people's willingness to perform CPR.

Currently, the American Heart Association (AHA) has a specific education program for adolescents. In Taiwan, CPR education has been provided for high-school students for years. To extend the benefits of such training, we started a new CPR training program in Taiwan for adults, and it revealed noninferior results to the conventional CPR training program [14]. However, little is known regarding the ability and learning effectiveness of school students who attend such basic life support (BLS) and AED courses. A European group named Kids Save Lives has claimed that training school children in CPR is highly effective, and 12 years is the suitable age to start teaching cardiac compression [15]. However, CPR quality is highly related to the body mass index (BMI) and exercise habits in EMTs [16]. Whether current adult CPR teaching programs are suitable for adolescents and whether these younger students can achieve the same CPR effectiveness as adults remain unknown. Therefore, the purpose of this study was (1) to evaluate the CPR effectiveness of both adolescents (12 years old) and adults who undergo the same course related to BLS and AED in the same environment and (2) to prove that the current adult BLS course is suitable for adolescents.

## 2. Method

### 2.1. Study Design, Setting, and Participants

This retrospective observation study was approved by the Chang Gung Memorial Foundation Institutional Review Board (approval number: 202000464B0). We extracted data from the database of an education program (IGOGO) and considered training courses between January 2018 and July 2018. The extracted data had to meet inclusion criteria, including students having the same training date and classroom, and participants were subsequently divided into an elementary sixth-grade students (elementary group) and adult group. The purpose of the training program was to promote the long-term implementation of CPR teaching combined with the use of AED for the public. To evaluate learning effectiveness in both elementary school students and adults, we selected participants who did not receive any CPR training for at least 1 year prior to taking this training course. Participants who were unable to kneel to perform CPR and those who were pregnant were excluded. A total of 308 participants were analyzed in the study, including sixth-grade students and teaching staff, security guards, and volunteers.

### 2.2. Education Course

IGOGO in Taiwan has been offering courses for many years. We use a standard 90-minute BLS training program, which is similar to the AHA course. The AHA program is a 90-minute, instructor-led, and classroom-based training program that employs the practice-while-learning format. The learning content includes an introduction to relevant laws, the purpose of CPR and AED, chains of survival, demonstration of the adult BLS sequence, CPR with AED use, and hands-on compression-only adult CPR.

The IGOGO program is taught by emergency physicians with BLS instructor qualifications who are assisted by nurses and doctors. The ratio of participants to manikins to instructor is 8 : 4 : 1. Sensor-equipped manikins (Resusci Anne with QCPR, Laerdal Medical AS, Norway) were used in the 2-minute hands-on practice in both groups. Each course consisted of a 60-minute CPR teaching video with practice, 20 minutes of instruction related to AED operation, and a 10-minute discussion concerning the legal aspect of bystander CPR in Taiwan.

### 2.3. Data Collection

Data collection focused on training program-specific data and the demographic data of participants. We also obtained informed consent from all participants and removed any personally identifiable information. We compiled course-related information, including basic student and adult data (which contain age, weight, height, gender, previous exercise habits, whether there is any previous CPR learning experience, and when was the last learning experience), pretest and posttest (e.g., knowledge of CPR and AED) results, skill tests, and CPR willingness questionnaire, into a database [17] (Appendix 1 in Supplementary Materials). All questions in the written test were formulated by staff of the Taiwan Society of Emergency Medicine, Emergency Medical Services Department. We assessed learning effectiveness in several manners. We assessed CPR and related knowledge by using a written test, which contained 15 multiple-choice questions with a maximum score of 100 (Appendix 2 in Supplementary Materials). CPR performance was evaluated in two aspects: manikin feedback and examiner evaluation. Objective data, including compression depth, compression rate, and full chest recoil, were recorded and collected from the feedback manikin. We followed the updated 2015 AHA Guidelines for CPR and Emergency Cardiovascular Care, in which high-quality CPR is defined as follows: (1) a compression rate of 100–120 beats per minute (bpm), (2) a compression depth of 5–6 cm, and (3) full chest wall recoil. Examiners rated participants' performance individually. Examiners assessed how well participants followed the BLS sequence in terms of skills on the checklist—from verifying scene safety to AED use (Appendix 3 in Supplementary Materials). We count each pass step as one point and fail step as zero points (total scores are 8). Ventilation was not included in this educational program because compression-only CPR is the current recommendation.

### 2.4. Outcome Measures

The purpose of this study was to compare CPR effectiveness between elementary students and adults in the same setting (i.e., learning subject and environment). The primary outcomes were CPR quality (a compression rate of 100–120 bpm, a compression depth of 5–6 cm, and full chest wall recoil) and passing or failing the following skill examination parameters: (1) confirm safety, (2) check consciousness, (3) call for help, (4) check breathing status, (5) CPR location, (6) CPR posture, (7) AED operation, and (8) AED pad location. The secondary outcome was the posttraining written test, questionnaire of CPR willingness, and total scores of skill examination (Appendices 1 and 2 in Supplementary Materials).

### 2.5. Statistical Analysis

Categorical variables were compared using the chi-squared test and are presented as numbers and percentages. Continuous variables are presented as means and standard deviations, and Student's *t*-test was used to compare the difference between two groups. The significance level *α* was set at 0.05. The data were analyzed using IBM SPSS Statistics (version 25.0 for Windows; IBM Corp., Armonk, NY, USA).

## 3. Results

**Figure 1:**
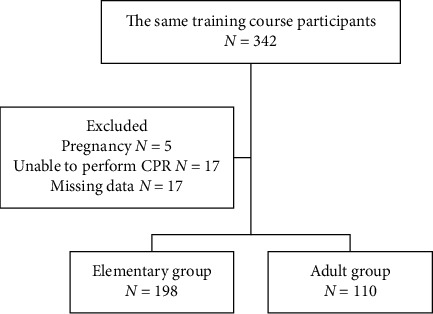
Flowchart. *N*: number; CPR: cardiopulmonary resuscitation.

In total, 342 people participated in the training program including 210 students and 132 school staff (Figure 1). We excluded those who were pregnant, were unable to adequately perform CPR, or had incomplete information; thus, 308 participants were eligible for analysis. Among them, 198 were elementary school students and 110 were school staff.  Table 1 lists the demographic statistics of the study. The mean age of elementary students was 11.8 years, and that of adults was 37.3 years. Females accounted for approximately half of the elementary group and 69% of the school staff. The two groups differed in terms of BMI, sport habits, and CPR learning experience.

**Figure 2:**
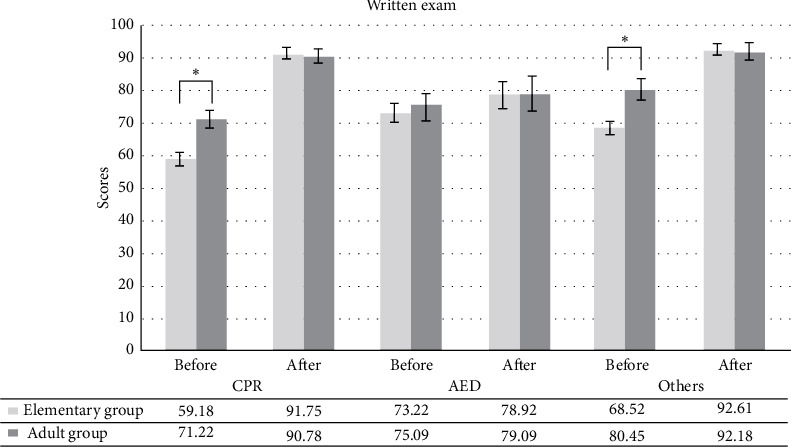
Difference of pre-/post-written exam after training. Data are expressed as score.  ^*∗*^indicates a significant difference. CPR: cardiopulmonary resuscitation; AED: automated external defibrillator.

The elementary group and adult group scored no difference in posttest (elementary group = 89.77; adult group = 91.62; *p*=0.064; Table 2). Regarding CPR quality, the elementary group achieved, on average, 114 bpm, a full chest recoil rate of 75.7%, and a compression depth of 4.68 cm. The adult group achieved, on average, 113 bpm, a full chest recoil rate of 77.2%, and a compression depth of 5.22 cm. In terms of CPR quality parameters, no significant differences were observed between the two groups (Table 2).  Figure 2 reveals a considerable improvement in both groups after taking the 90-minute course. The elementary and adult groups differed significantly in the pretest, except for AED knowledge. However, it showed no statistical difference in all three items (CPR, AED, and others) after the course.

Regarding skill items shown in Table 3, elementary students performed CPR as effectively as adults in almost all skills including verifying scene safety, checking consciousness, calling for help, CPR location, CPR posture, AED operation, and AED pad location. However, a significant difference was observed in checking breathing status and total scores: the success rate in the elementary group was 71%, whereas it was 86% in the adult group (*p*=0.003), and total scores were 6.18 (1.284) and 6.61 (1.342), respectively. We also investigated willingness to perform CPR. The results revealed no difference in willingness to perform hands-only CPR on an acquaintance but a significant difference in willingness to do so on a stranger (elementary group = 51%; adult group = 39.1%; *p*=0.045; Table 4). The three major reasons why participants were unwilling to perform CPR on either an acquaintance or a stranger are fearing doing further harm, fearing performing CPR incorrectly, and being unwilling to perform cardiac compression.

## 4. Discussion

Our results revealed that, after undergoing the same training program, sixth-grade elementary students could perform CPR as effectively as adults could in three aspects, namely, compression depth, compression rate, and full chest recoil. However, elementary students struggled to meet the AHA high-quality benchmark for compression depth. In a study of school children aged 7–14 years, chest compression depth was highly correlated with children's age, weight, height, and BMI [18]. In a UK study that divided children into three groups, namely, 9-10, 11-12, and 13-14 years, only the 13-14-year-old group could perform chest compression just as well as adults [19]. Therefore, when teaching high-performance CPR to elementary school students, instructors should focus on the knowledge and process, instead of requiring students to reach the depth mentioned in the AHA guidelines because of the children's limited ability to perform chest compression. Notably, the children and adults differed significantly in terms of checking breathing status despite following the same learning program and checklist; previous similar studies have not noted this disparity. Hence, instructors should devote special attention to teaching elementary students how to check breathing status in the future.

Currently, no regulations or rules stipulate when to implement CPR education in school settings in Taiwan. However, such programs are typically introduced in high school. According to the Kids Save Lives group in Europe, CPR training should start from the age of 12 years or even younger; moreover, annual CPR refresher courses should be offered [20–22]. Training can be offered successfully with a low-cost manikin and equipment by either medical professionals or educated teachers [23]. Studies have reported that children aged 10–12 years have the same CPR effectiveness as adults do [22, 24]. Our results corroborate those findings. In the written test, both children and adults exhibited considerable improvement between the pretest and posttest. Moreover, the 12-year-old elementary students had sufficient ability to undergo current CPR education and could comprehend and apply the training as well as adults could. Notably, AED knowledge in the pretest did not differ significantly between the two groups. This finding may be attributed to the widespread public-service announcements related to AED and also the implementation of AED in the school where our study was conducted. Therefore, as AED installations become more common, both promotion of AED and people's knowledge of it will increase.

Children included in this study could complete the CPR checklist items just as well as adults for almost all elements. However, children's performance in checking breathing status was inferior to that of adults; they either forget this element or performed it incorrectly. Checking breathing status is also a difficult task for adults: in conventional CPR education, adults struggle to differentiate bradypnea or agonal breathing. Thus, challenges faced by adolescents in CPR education might be understandable. A systematic review revealed that, by the age of 11-12 years, children can establish whether a victim is conscious and breathing normally [25]. We suggest to use virtual reality, augmented reality, and gamified learning to enhance the effectiveness of learning in the education program [26]. Results for AED pad location in the current study also merit further attention. Although the two groups did not differ significantly, the numerical difference was quite large (71.72% vs. 86.36%). Thus, children may easily confuse the correct placement of an AED. For CPR education to have the same effectiveness in both children and adults, we argue that such programs should focus more on checking breathing status and AED pad location. More crucially, children should be taught to activate the local emergency medical services system earlier because most cardiac arrest cases occur in residential areas [3, 4].

Whether children can perform CPR adequately still requires further study. In a study in a rural area of Taiwan, a 50-minute CPR/AED course signiﬁcantly improved the knowledge of adolescents and empowered them to be willing to perform CPR if necessary [27]. Our study revealed the same trend. Children seem to be more willing to perform CPR on either a stranger or an acquaintance. Therefore, early promotion of CPR education is valuable for elementary school students. As a result of such training, the bystander CPR rate might increase in the future. In addition, in our questionnaire, children reported anxiety regarding performing CPR on strangers mostly because they fear causing harm if they perform it incorrectly; adults were more concerned with the legal aspect and contracting an infectious disease [28, 29]. Such reluctance may be overcome with sufficient training and by boosting children's confidence.

To sum up, we advocate offering the current 90-minute BLS and AED course to sixth-grade elementary students because they can understand the content and perform CPR as effectively as adults can. More widespread training may increase bystander CPR rates and subsequently result in survival benefits for those experiencing OHCA worldwide.

## 5. Limitation

This retrospective study was conducted with data from the IGOGO database; as a result, it has some limitations. First, we evaluated the performance in an educational setting; the current results may not represent children's actual performance when facing a real emergency situation. Finally, this study did not track the learning status; hence, we could not assess whether children's CPR abilities are maintained over time; this aspect may be considered in future research.

## 6. Conclusion

In summary, we revealed that sixth-grade elementary students' performance in CPR and AED was similar to that of adults after completing the current 90-minute course. Because children are more willing to perform CPR than adults are, we strongly advocate offering CPR and AED courses to 12-year-old children, and these courses should emphasize checking the victim's breathing status.

## Figures and Tables

**Table 1:** Demographic population.

	Elementary group	Adult group
Numbers	198	110
Age, years (SD)	11.8 (0.46)	37.27 (10.22)
Height, cm (SD)	152.8 (7.32)	160.77 (7.36)
Weight, kg (SD)	41.9 (8.70)	60.75 (11.43)
BMI (SD)	17.83 (3.018)	23.85 (3.357)
Female (%)	101 (51.01%)	76 (69.09%)
Sport habits (%)	154 (77.78%)	51 (46.36%)
<1 hour	122 (61.62%)	28 (25.45%)
>1 hour	32 (16.16%)	23 (20.91%)
CPR learning experience (%)	64 (32.32%)	94 (85.45%)
1∼2 years	48 (24.24%)	72 (65.45%)
>2 years	7 (3.54%)	10 (9.09%)
Unknown	9 (4.54%)	12 (10.91%)
Type of CPR (%)		
Hands-only CPR	55 (27.78%)	71 (64.54%)

Data are expressed as mean (SD) or *n* (%). cm: centimeter; kg: kilogram; SD: standard deviation; BMI: body mass index; CPR: cardiopulmonary resuscitation.

**Table 2:** Assessment of the cardiopulmonary resuscitation training course.

	Elementary group	Adult group	*p* value
*Post-written test score (SD)*	89.77 (8.28)	91.62 (8.68)	0.064
CPR (SD)	91.75 (12.00)	90.78 (12.33)	0.499
AED (SD)	78.92 (26.67)	79.09 (27.78)	0.872
Others (SD)	92.61 (11.66)	92.18 (13.57)	0.77

*Quality assessment*			
BPM (SD)	114.15 (20.556)	113.17 (13.03)	0.18
Recoil (SD)	75.7 (32.0)	77.2 (31.0)	0.33
Depth (SD)	4.68 (0.95)	5.22 (0.81)	0.12

Data are expressed as mean (SD). SD: standard deviation; CPR: cardiopulmonary resuscitation; AED: automated external defibrillator; BPM: beats per minute.

**Table 3:** Skill examination parameters of cardiopulmonary resuscitation.

	Elementary group	Adult group	*p* value
Numbers	198	110	
Confirm safety	123 (62.12%)	72 (65.07%)	0.561
Check consciousness	172 (86.87%)	92 (83.64%)	0.437
Call for help	163 (82.32%)	94 (85.45%)	0.479
Check breathing status	142 (71.72%)	95 (86.36%)	0.003^*∗*^
CPR location	154 (77.78%)	91 (82.73%)	0.302
CPR posture	153 (77.27%)	92 (83.64%)	0.185
AED operation	160 (80.81%)	95 (86.36%)	0.216
AED pad location	156 (78.79%)	96 (87.27%)	0.064
Total scores	6.18 (1.284)	6.61 (1.342)	0.006^*∗*^

Data are expressed as mean (SD) or *n* (%).  ^*∗*^indicates a significant difference. SD: standard deviation; CPR: cardiopulmonary resuscitation; AED: automated external defibrillator.

**Table 4:** Willingness to perform CPR after training.

	Elementary group	Adult group	*p* value
*Willing to perform CPR on the associate*			
Yes	188 (94.95%)	99 (90.0%)	0.099
No	10 (5.05%)	11 (10.0%)	

*Reasons of unwillingness*			
Afraid of doing further harm	10	5	
Afraid of doing CPR incorrectly	10	7	
Unwilling to perform cardiac compression	9	4	
Afraid of legal issues	7	11	
Others	4	2	

*Willing to perform CPR on a stranger*			
Yes	101 (51.01%)	43 (39.09%)	0.045^*∗*^
No	97 (48.99%)	67 (60.91%)	

*Reasons of unwillingness*			
Afraid of doing further harm	43	37	
Afraid of doing CPR incorrectly	35	28	
Unwilling to perform cardiac compression	31	24	
Afraid of legal issues	27	8	
Others	23	21	

Data are expressed as *n* (%) or *n*.  ^*∗*^indicates a significant difference. CPR: cardiopulmonary resuscitation.

## Data Availability

The data that support the findings of this study are available from the corresponding author upon reasonable request (e-mail: ngowl@ms3.hinet.net).

## Supplementary Materials

Supplementary Materials CPR willingness questionnaire.
