# Supplementary material for: Learning Effectiveness Assessment between Primary School Students and Adults in Basic Life Support Education
Source: Emerg Med Int. 2021 Feb 24;2021:5579402. doi: 10.1155/2021/5579402 (PMC7929662; doi:10.1155/2021/5579402)
Supplement: Supplementary Materials — CPR willingness questionnaire. [file 5579402.f1.docx]

Appendix 1.

**Chang Gung Medical Foundation CPR+AED Training Program**

**CPR willingness Questionnaire**

When someone is unconscious and has no spontaneous breathing or heartbeat and has been reported to 911, CPR should be performed immediately according to the recommendations of American Heart Association (AHA).Please answer the following questions

1. If this person were a stranger, would you be willing to perform conventional CPR (mouth-to-mouth resuscitation) on her (him) immediately?

□ Yes (skip to question5) □ No

2. If you are not willing to perform CPR on a stranger, what is the reason(s)? (select all that apply)

□ (1) Concern about doing more harm to the person

□ (2) Concern that CPR cannot be performed correctly

□ (3) Concern about legal issues

□ (4) Not willing to perform mouth-to-mouth resuscitation

□ (5) Do not want to perform CPR

□ (6) Other, please specify the reason(s):

3. From the reasons listed above, select the option that is the most important reason that you do not want to perform CPR.

□(1) □(2) □(3) □(4) □(5) □(6)

4. If you select Option (4) in Q2 and you are not required to perform mouth-to-mouth resuscitation, would you be willing to perform chest compression on a stranger?

□ Yes □ No

5. If this person were a family member or an associate, would you be willing to perform conventional CPR on her (him) immediately?

□ Yes □ No

6. If you are not willing to perform CPR on a family member or an associate, what is the reason(s)? (select all that apply)

□ (1) Concern about doing more harm to the person

□ (2) Concern that CPR cannot be performed correctly

□ (3) Concern about legal issues

□ (4) Not willing to perform mouth-to-mouth resuscitation

□ (5) Do not want to perform CPR

□ (6) Other, please specify the reason(s):

7. From the reasons listed above, select the option that is the most important reason that you do not want to perform CPR.

□(1) □(2) □(3) □(4) □(5) □(6)

8. If you select Option (4) in Q2 and you are not required to perform mouth-to-mouth resuscitation, would you be willing to perform chest compression on a family member or an associate?

□Yes □ No

Appendix 2.

**Pretest Chang Gung Medical Foundation CPR+AED Training Program**

**Written Test Paper**

**Participant Unit： Participant Name： Date： year month day**

※Please complete the paper before lesson starts.

1、( ) Which one of the following is the most critical element for the general public to confirm that a patient needs CPR?
(1) Not waking up
(2) Severe chest pain
(3) Non-stop convulsions
(4) Unconscious and not breathing normally

2、( ) What is the purpose of using AED?

(1) To input external energy to the patient's heart so the heart can work again

(2) To massage the patient's chest muscles with electricity to relieve discomfort

(3) To restore the heart’s beating after defibrillate the patient's ventricular fibrillation

(4) To replace CPR

3、( ) You witness an adult suddenly collapsed, has no response upon shouting and tapping, and is not breathing normally. In order to save his life, which of the following is the most appropriate action？

(1) Stand aside and see if he will be better afterward
(2) Immediately start chest compressions

(3) Shouts for help, activates emergency medical response system, and ask someone to get AED

(4) Leave the scene

4、( ) According to the 2015 version of the American Heart Association CPR and ECC guidelines, what is the most appropriate chest compression depth for adults？

(1) 3 to 4 cm

(2) 4 to 5 cm

(3) 5 to 6 cm

(4) 6 to 7 cm

5、( ) According to the 2015 version of the American Heart Association CPR and ECC guidelines, what is the most appropriate chest compression rate for adults？

(1) 90 to 100 times per minute

(2) 100 to 110 times per minute

(3) 100 to 120 times per minute

(4) 110 to 120 times per minute

6、( ) Which of the following is a requirement for high-quality CPR?

(1) More than 80 compressions per minute

(2) Compression depth 4-5 cm

(3) Try to avoid interruptions

(4) For adults and children, the first aid compression-ventilation ratio is 30:2 for 1 or 2 rescuers

7、( ) The correct position to perform CPR chest compression is？
(1) The sternum in the middle of the line between the two nipples

(2) Go up below the xiphoid process

(3) Upper middle sternum

(4) Right above the left heart

8、( ) The implementation of chest compressions emphasizes that interruption should be avoided. If there is an interruption, it should not exceed how long？

(1) 5 seconds

(2) 10 seconds

(3) 15 seconds

(4) 20 seconds

9、( ) How to give the fallen sudden cardiac arrest patient a chance of survival？

(1) Start the emergency medical response system as soon as possible

(2) Start CPR as soon as possible

(3) Shock the victim who has "shockable rhythm" as soon as possible

(4) All of the above

10、( )After how many minutes of CPR we should pause to reassess the victim’s responsiveness and breathing？

(1) 4 minutes

(2) 3 minutes

(3) 2 minutes

(4) 1 minute

11、( ) The Good Samaritan law provides exemption protection for temporary first-aid providers. Which law does it come from?

(1) Criminal law

(2) Civil law

(3) Physician Law

(4) Emergency Medical Aid Act

12、( ) Which of the following is not a condition for stopping CPR？

(1) Ambulance arrival

(2) Victim start to have response

(3) Has performed five cycles of chest compressions with rescue breath

(4) Rescuer feels sick

13、( ) To effectively perform external cardiac massage (also known as chest compression), which of the following are the action that the rescuer must do?

➀ Allow the chest to return to the its original position ➁ Make chest compression time equal to relaxation time ➂ Keep your hands in the correct position ➃ Remove your hands after each chest compression to confirm the compression position

(1) ➀+➁+➃

(2) ➀+➁+➂

(3) ➁+➂

(4) ➀+➂

14、( ) Which of following is not a description about AED (automated external defibrillator)？

(1) AED is not recommended for use in infants and children under 8 years of age

(2) It is recommended that the police must be trained to learn how to use AED

(3) The so-called PAD (public access defibrillation) means that the AED needs to be placed in a public area with a dense population of possible sudden cardiac arrest

(4) AED can be used in patient who has an implantable pacemaker when he/she has ventricular fibrillation (Vf)

15、( ) There are four common steps in the operation of AED (universal AED common steps) as follows, what is the correct order of the steps?

➀ Analyze heart rhythm ➁ Attach pads ➂ Press the shock button ➃ Turn on the power

(1) ➃🡪➀🡪➁🡪➂

(2) ➁🡪➀🡪➃🡪➂

(3) ➃🡪➁🡪➀🡪➂

(4) ➁🡪➃🡪➀🡪➂

**Post-test Chang Gung Medical Foundation CPR+AED Training Program**

**Written Test Paper**

**Participant Unit： Participant Name： Date： year month day**

※Please complete the paper after the course

1、( ) If you find your colleague suddenly collapse at the dining table, how should you response？

(1) Immediately pat his back with your hand

(2) Immediately let your colleague lie down and give Hamrick First Aid

(3) Immediately perform oral finger sweeping method

(4) Immediately confirm the patient's consciousness and breathing, if unconscious and not breathing, quickly call 119

2、( ) What should the rescuer do when using an AED for electric shock？

(1) Touch the patient

(2) Use hands to protect the patient from injury during electric shock

(3) Check the pulse and immediately perform CPR after delivering shock as recommended by AED

(4) Pad for adults can be used on children when a suitable pad cannot be found

1. ( ) What is the purpose of CPR chest compression?

(1) For exercise

(2) To ensure that the patient’s blood flow maintenance

(3) To test your balancing

(4) To make sure that the patient’s lungs are function well

4、( ) What is the rate of CPR chest compression？

(1) 60~80 per minute

(2) 100~120 per minute

(3) 140~160 per minute

5、( ) What is the compression depth for adult？

(1) 5~6 cm

(2) 7~8 cm

(3) 9~10 cm

6、( ) Which one of the following is not the definition of high-quality CPR for adults？

(1) Sufficient number and depth of chest compressions must be provided

(2) Allow full chest recoil after each compression

(3) Reduce the interruption of chest compressions

(4) The depth of compression is 4-5 cm

7、( ) Where is the position for cardiac massage in adult CPR？

(1) Two fingers below the xiphoid

(2) Midpoint of nipple line

(3) One finger below the nipple line next

(4) Mid sternum

8、( ) High-quality chest compressions emphasize "do not interrupt". The interruption should not be exceed for how long？

(1) Can't stop at all

(2) 10 seconds

(3) 20 seconds

(4) Case dependent, there is no unified approach

9、( ) You witness an adult suddenly collapsed, unresponsive upon calling him loudly and tapping, and not breathing normally. In order to save his life, which of the following is the most appropriate action？

(1) Stand aside and see if he will be better afterward

(2) Immediately start chest compressions

(3) Shout for help, activate the emergency medical response system, and ask someone to get the AED

(4) Leave the scene

10、( ) How long is the “golden-time” for initiating a CPR？

(1) 1-3 minutes

(2) 4-6 minutes

(3) 7-9 minutes

(4) 10-12 minutes

11、( ) Which laws in our country provide exemption protection for temporary first-aid provider？

(1) Criminal law

(2) Civil law

(3) Physician Law

(4) All of the above

12、( ) CPR need not to be performed in which of the following situation?

(1) Limbs are not stiff and have temperature

(2) Pupil constriction

(3) Pupil dilation

(4) With a do-not-resuscitate order

13、( ) The rescuer’s posture will affect the effectiveness of CPR. What are the proper hand placement?

➀ Shoulder above the patient's chest ➁ Maintain the correct position of the heel of the hand and keep the arms straight during chest compressions ➂ Use your body weight instead of just pushing forward with both hands

(1) ➀+➁

(2) ➀+➁+➂

(3) ➀+➂

(4) ➁+➂

1. ( ) Which of the following statement is wrong when using an AED?

(1) If the patient has too much chest hair, consider shaving with a razor

(2) Do not use AED in water

(3) Patients >1 year old can use AED

(4) If the patient has implantable pacemaker, AED should not be used

15、( ) Which of the following is wrong about AED？

(1) When the suitable electric shock pad cannot be found, the adult pad can be used on children

(2) Continue CPR and do not stop when the machine is analyzing the heart rate

(3) Do not touch the patient during shock

(4) After the shock, continue CPR for 2 minutes and then reassess heart rate

Appendix 3.

**Chang Gung Medical Foundation CPR+AED Training Program**

**CPR Practice (Skill Testing) Checklist**

Student Name:

Affiliation:

Date of Test： (MM/DD/YYYY)

Course Subject Evaluation Form (to be filled by the examiner)

| **Step** | **Item** | **PASS** | **FAIL** | | |
| --- | --- | --- | --- | --- | --- |
| 1 | Verbally confirm that the scene is safe |  |  | | |
| 2 | Checks responsiveness |  |  | | |
|  | Confirm response: tap the patient |  | | | |
|  | Confirm response: call the patient’s name loudly |  |  |  |  |
| 3 | Calls for help |  |  | | |
|  | Ask someone to call 911 |  | | | |
|  | Ask someone to get an AED |  |  |  |  |
| 4 | Checks breathing |  |  | | |
|  | Check for no breathing or agonal breathing |  | | | |
|  | Confirm that the patient has not breathed for 5-10 seconds |  |  |  |  |
| 5 | CPR position |  |  | | |
|  | Place the heel of your hand in the right place for chest compression: in the inter-nipple line | | | | |
| 6 | CPR posture |  |  | | |
|  | Place your other hand on top of the first hand |  | | | |
|  | Keep the CPR arm straight |  |  |  |  |
|  | Keep the CPR arm perpendicular to the body |  |  |  |  |
|  | Allow the student to continue chest compressions | | | | |
| The examiner says: “The AED arrives on scene.” | | | | | |
| 7 | AED operating |  | |  | |
|  | After arrival of the AED, turns on the power of AED |  | | | |
|  | Attaches the pads |  |  |  |  |
|  | Before pushing the “shock” button, shout “keep clear” |  |  |  |  |
| 8 | Confirm that the pads are placed in the correct place |  | | |  |
| End the Test | | | | | |

Examiner’s Signature:

Score:
